# Functional Depth Biomarkers Distinguish Lung Squamous Cell Carcinoma from Lung Adenocarcinoma

**DOI:** 10.21203/rs.3.rs-9347794/v1

**Published:** 2026-07-13

**Authors:** Sagnik Bhadury, Ashley P. Tsang, Dipankar Ray, Kiran Lagisetty, Arvind Rao

**Affiliations:** 1 Department of Computational Medicine and Bioinformatics, Michigan Medicine, University of Michigan, Ann Arbor, MI, USA; 2 Department of Radiation Oncology, University of Michigan, Ann Arbor, MI, USA; 3 Department of Surgery, University of Michigan, Ann Arbor, MI, USA; 4 Department of Biostatistics, University of Michigan, Ann Arbor, MI, USA; 5 Department of Biomedical Engineering, University of Michigan, Ann Arbor, MI, USA

## Abstract

Lung squamous cell carcinoma (LUSC) and lung adenocarcinoma (LUAD) exhibit fundamentally distinct pathway coordination architectures. We developed a framework integrating pathway activity inference from spatial transcriptomics data, spatial proximity based network construction, and functional depth analysis across 996 TCGA patients. Applying Fraiman-Muniz depth statistics, we generated two representations: Population Referenced Depth quantifies typicality relative to population distributions, while Patient Referenced Depth assesses within-patient network organization. Random forest classification revealed that Patient Referenced Depth marginally outperforms population comparisons, achieving test AUC of 0.768. We focus on bidirectional interaction patterns obtained from spatial interaction networks of pathways. Multi-method feature integration identified three mechanistic frameworks distinguishing subtypes: myeloid orchestrated immune coordination (JAK-STAT ↔ TNF*α* dominates LUAD through SPP1^+^ macrophage niches), mutation driven pathway rewiring (TP53 mutations create ecosystem-wide reorganization in LUAD but homogeneous baseline in LUSC), and hypoxia-hormone microenvironment programming (peripheral LUAD tumors coordinate fluctuating hypoxia with angiogenesis while central LUSC tumors integrate chronic hypoxia with death receptor regulation). We identified five novel LUSC-enriched interactions (Androgen ↔ TRAIL, EGFR ↔ Estrogen, EGFR ↔ TNF*α*, Hypoxia ↔ TRAIL, TGF*β* ↔ TRAIL) that remain mechanistically uncharacterized, revealing critical knowledge gaps. This framework provides a statistically principled approach for extracting actionable biomarkers from biological networks with applications to precision oncology and therapeutic target identification.

## 1 Introduction

Lung adenocarcinoma (LUAD) and lung squamous cell carcinoma (LUSC) represent the two most common histological subtypes of non-small cell lung cancer, accounting for over 70% of lung cancer diagnoses^1^. These subtypes differ fundamentally in molecular architecture, anatomical origin, and therapeutic vulnerability. LUAD develops peripherally from type II pneumocytes or Clara cells and harbors actionable mutations in EGFR, KRAS, and ALK, enabling targeted therapeutic strategies^2^. LUSC arises centrally from bronchial epithelial cells and exhibits near-universal TP53 mutations (over 80% of cases) alongside SOX2 and TP63 amplifications driving squamous differentiation programs^3,4^. Despite well-characterized genomic differences, accurate discrimination remains challenging in poorly differentiated tumors and small biopsy specimens, with significant implications for treatment selection^5^.

Recent multi-omics profiling reveals that LUAD and LUSC exhibit fundamentally distinct pathway coordination architectures rather than isolated pathway differences. LUAD demonstrates coordinated JAK-STAT and TNF*α* signaling that maintains chronic inflammatory microenvironments mediated by SPP1^+^ macrophage niches^6–8^. Physical interaction between HIF-1*α* and STAT3 stabilizes HIF-1*α* and regulates approximately 30% of hypoxia-induced genes, creating bidirectional regulatory circuits that integrate oxygen sensing with inflammatory signaling^9,10^. LUSC exhibits dominant TNFR1-NF*κ*B signaling through the TNFR1-UBCH10 axis, with TNF receptor engagement inducing nuclear translocation of NF-*κ*B subunits that drive dedifferentiation and metastasis programs^11^. Anatomical differences in tumor location create distinct microenvironmental pressures: peripheral LUAD tumors experience fluctuating hypoxia with intermittent reoxygenation, while central LUSC tumors develop chronic stable hypoxia with necrotic cores^5^. These biological differences manifest as coordinated pathway interaction patterns that distinguish subtypes more robustly than individual gene mutations^12,13^.

Traditional differential gene expression approaches miss systems-level pathway coordination that characterizes cancer biology^14^. Pathway-based methods typically analyze pathways independently, missing critical interaction effects between signaling networks^15^. Network-based representations provide a natural framework where pathways constitute nodes and their coordinated activities define weighted edges^16^. Graph-theoretic approaches have identified modular pathway organization differences between LUAD and LUSC through weighted gene co-expression network analysis^17^, and graph attention networks enable molecular stratification with enhanced interpretability^18^.

A critical limitation of population-level biomarker approaches is that they compare patients to cohort-wide distributions, potentially missing patient-specific molecular contexts. N-of-1 precision oncology trials demonstrate that therapies matched to individual molecular profiles via personalized combination regimens improve outcomes compared to population-derived biomarkers, with matched patients achieving longer progression-free survival (6.4 versus 3.0 months) and overall survival (15.3 versus 4.7 months)^19,20^. This motivates assessing pathway dysregulation relative to each individual’s baseline signaling architecture rather than population norms^21,22^.

Functional data analysis provides principled methods for characterizing complex data structures^23^. Data depth quantifies how central or typical an observation is within a multivariate distribution, generalizing notions of rank to high-dimensional spaces^24^. The Fraiman-Muniz depth integrates pointwise depths across functional domains^25^, transforming complex functional objects into scalar features while preserving global structural information. Random forest-based feature selection handles high-dimensional genomic data effectively, offering superior stability and interpretability^26,27^. Combined with differential expression analysis and principal component analysis, random forests enable multi-method triangulation that enhances confidence in identified biomarkers^28^.

We developed a computational framework integrating pathway activity inference, network construction, functional data analysis, and machine learning to identify discriminative biomarkers distinguishing LUAD from LUSC. We constructed patient-specific pathway interaction networks by inferring activities for 14 cancer-related pathways from bulk RNA-seq data using PROGENY^29^ and quantifying pairwise pathway coordination through partial correlations derived from spatial proximity patterns. We computed two depth-based feature representations: Population Referenced Depth quantifies population-level typicality, while Patient Referenced Depth captures within-patient network organization. Random forest classification combined with systematic rule extraction, co-occurrence network analysis, and multi-method feature integration identified biologically interpretable pathway interaction signatures.

Patient Referenced Depth outperformed Population Referenced Depth in classification accuracy (70.80% versus 69.10%) with balanced sensitivity and specificity, demonstrating that internal signaling architecture more robustly captures subtype-specific molecular features than population comparisons. Throughout this manuscript we have utilized the notation ↔ to encapsulate the bi-directional regulatory proximity based interactions among the pathways. We identified JAK-STAT ↔ TNF*α* coordination as the dominant discriminative feature in LUAD, NF*κ*B ↔ TNF*α* interactions characteristic of LUSC, and Hypoxia ↔ JAK-STAT crosstalk reflecting subtype-specific microenvironmental adaptation. These signatures align with mechanistic studies demonstrating HIF-1*α*-STAT3 interaction^9^, TNFR1-UBCH10 axis activation^11^, and SPP1^+^ macrophage niches in LUAD^7,8^. We nominate five LUSC-enriched pathway interactions (Androgen ↔ TRAIL, EGFR ↔ Estrogen, EGFR ↔ TNF*α*, Hypoxia ↔ TRAIL, TGF*β* ↔ TRAIL) that remain mechanistically uncharacterized, revealing critical knowledge gaps in LUSC biology with therapeutic implications. Our framework provides a statistically principled approach for extracting interpretable biomarkers from biological networks with applications to immunotherapy response prediction and therapeutic target identification.

## 2 Results

### 2.1 Study Design and Data Characteristics

We analyzed in silico spatial transcriptomics data from 996 TCGA lung cancer patients, comprising 525 LUAD and 471 LUSC cases. After stratified splitting, the training set contained 698 samples (330 LUSC, 368 LUAD) and the test set had 298 samples (141 LUSC, 157 LUAD). Using PROGENY^29^, we inferred activities for 14 cancer related pathways (Androgen, Estrogen, Hypoxia, JAK-STAT, MAPK, NF*κ*B, p53, PI3K, TGF*β*, TNF*α*, Trail, VEGF, WNT, EGFR) across spatial locations within each spatial transcriptomics image. We constructed patient specific pathway interaction graphs by computing pathway proximity to each spatial location using G-cross^30^ and applying partial correlations to obtain the graph structure, yielding 91 unique edges per patient after soft imputation and symmetrization (for more details see Methods).

**Figure 1.**
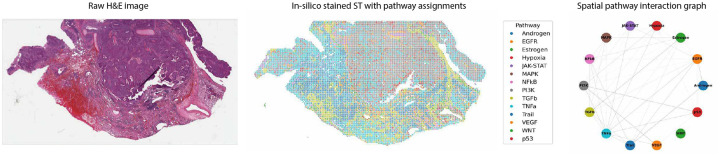
Construction of spatial pathway interaction graphs from TCGA histopathology images. The analytical pipeline consists of three stages: (*Left*) Raw hematoxylin and eosin (H&E) stained histopathology slide from a TCGA lung cancer sample, showing the original tissue architecture. (*Center*) In-silico spatially resolved transcriptomics map reconstruction where locations are classified into 14 cancer pathways using gene expression. Hexagonal grids represent spatial units, colored according to the dominant pathway at that location. This spatial mapping reveals heterogeneous pathway activity distributions across the tumor microenvironment. (*Right*) Spatial pathway proximity graph where nodes represent individual pathways (color-matched to center panel) and edges represent spatial co-occurrence relationships. Edge presence indicates that two pathways are spatially proximate or co-active in tissue regions.

Figure 1 illustrates our three-stage pipeline for constructing spatial pathway interaction graphs from TCGA histopathology images. Beginning with raw hematoxylin and eosin (H&E) stained tissue slides, we generated in-silico spatial transcriptomics representations by mapping gene expression patterns to spatial locations within the tumor. Each spatial location was assigned to one of 14 cancer-relevant pathways based on dominant gene expression signatures. These pathways encompass key oncogenic processes including growth factor signaling (Androgen, EGFR, Estrogen), stress and inflammatory responses (Hypoxia, JAK-STAT, MAPK, NF*κ*B), proliferation and angiogenesis regulators (PI3K, TGF*β*, TNF*α*, Trail, VEGF, WNT), and tumor suppressor activity (p53). The spatial mapping reveals heterogeneous distributions of pathway activities across the tumor microenvironment, reflecting intratumoral functional diversity.

From the spatially resolved pathway assignments, we constructed pathway interaction graphs where nodes represent individual pathways and edges represent spatial co-occurrence relationships. An edge between two pathways indicates that they are spatially proximate or co-active in neighboring tissue regions. This graph representation captures the functional organization of the tumor, encoding which pathways tend to be active together and how they are spatially coordinated. The resulting graph adjacency matrix for each patient serves as input for our functional data analysis where the nodes are the pathways and the edges encapsulate the bi-directional regulatory proximity based interactions among the pathways. These bi-directional interactions are explained through the notation “↔”. To this end, each of the 14 pathways corresponds to a node, yielding 142=91 unique pairwise interactions (edges). These 91 edge weights, extracted from the upper triangle of the symmetric adjacency matrix, constitute the functional data vectors analyzed in subsequent depth-based classification models. Each patient is thus characterized by a unique pathway interaction network topology that reflects their tumor’s spatial-functional architecture.

### 2.2 Functional Depth Features Capture Different Aspects of Network Organization

We computed two complementary depth-based feature representations. Population Referenced Depth quantifies how typical each patient’s pathway interaction strength is relative to the population distribution for that specific interaction, treating each of the 91 edges as a functional object across patients. Patient Referenced Depth assesses how typical each pathway interaction is within a patient’s own network, providing an internal reference frame. Critically, for Population Referenced Depth, we performed depth computation exclusively on training data and scored test samples against the training distribution to prevent data leakage.

These depth measures capture fundamentally different biological questions. Population Referenced Depth identifies patients whose molecular configurations deviate from population norms, potentially revealing outlier interaction patterns characteristic of disease subtypes. Patient Referenced Depth characterizes the internal organization of each patient’s pathway network, revealing which interactions are unusually strong or weak relative to that individual’s overall signaling architecture. Visual inspection of depth distributions revealed notable differences between LUAD and LUSC for several pathway interactions, with Population Referenced Depth features showing broader dynamic ranges. (Details on Depth calculations are provided in the Methods Section)

### 2.3 Within Patient Network Depth Provides Better Subtype Discrimination

**Figure 2.**
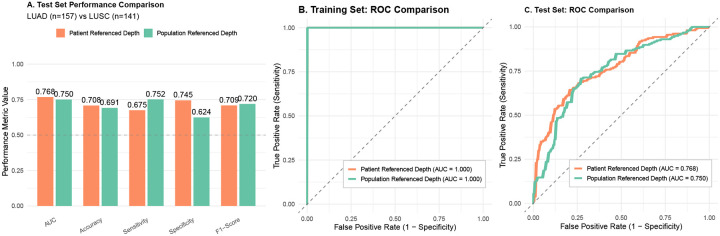
Random forest classification performance comparison between Population Referenced Depth and Patient Referenced Depth models. (A) Test set performance metrics show Patient Referenced Depth achieves higher specificity (74.5% vs 62.4%) while Population Referenced Depth favors sensitivity (75.2% vs 67.5%). Both models achieve comparable AUC (0.768 vs 0.750). (B) Training set ROC curves demonstrate perfect separation (AUC = 1.000) for both approaches. (C) Test set ROC curves confirm robust generalization with Patient Referenced Depth showing marginally superior discriminative performance.

Random forest classification revealed distinct performance profiles for the two depth approaches (Table 1, Figure 2). The Patient Referenced Depth model (within patient) achieved superior test set performance with AUC of 0.768, accuracy of 70.8%, sensitivity of 67.5%, and specificity of 74.5%. The Population Referenced Depth model (across patient) yielded comparable test AUC of 0.751 with accuracy of 69.1%, but exhibited a different sensitivity-specificity trade-off profile with higher sensitivity (75.2%) and lower specificity (62.4%).

The contrasting performance profiles suggest these approaches capture fundamentally different biological information. Population Referenced Depth prioritizes sensitivity over specificity, capturing a broader range of LUAD cases but with reduced precision in LUSC identification. In contrast, Patient Referenced Depth achieves more balanced performance with superior specificity, suggesting that within patient network organization provides more robust discrimination of coordinated signaling patterns that distinguish subtypes. The slight improvement in overall accuracy (70.8% versus 69.1%) combined with better specificity and AUC indicates that Patient Referenced Depth captures more discriminative features of subtype-specific pathway coordination. Out of bag error estimates closely matched test performance for both models, indicating stable generalization beyond the specific train-test split. Though we present both models, and emphasize that both models capture distinct scenarios, we focus on Patient Referenced Depth given its superior discriminative properties and clinical implications for precision medicine applications.

### 2.4 Decision Rule Analysis Reveals Interpretable Classification Logic

**Figure 3.**
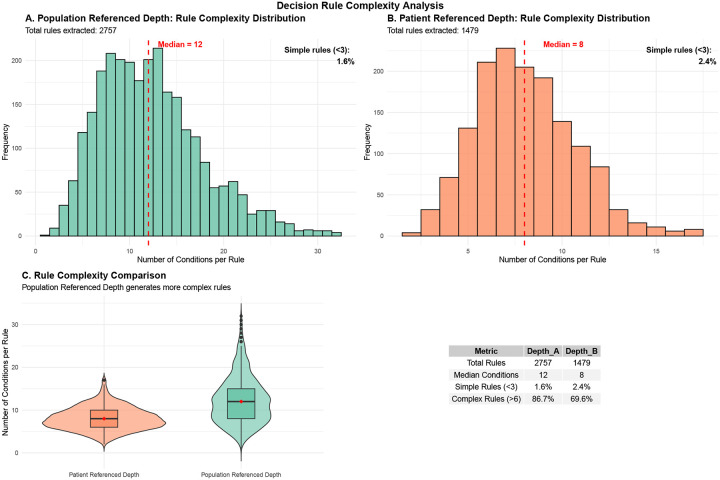
Decision rule complexity analysis from random forest models. (A,B) Distribution of rule complexity measured by number of conditions per rule. Population Referenced Depth generates more complex rules (median = 12 conditions) compared to Patient Referenced Depth (median = 8 conditions), with only 1.6% and 2.4% simple rules (< 3 conditions) respectively. (C) Violin plots with overlaid boxplots confirm Population Referenced Depth requires substantially more complex decision boundaries. Red diamonds indicate median values. (D) Summary statistics table shows Population Referenced Depth (Depth A) extracted 2757 total rules with 86.7% classified as complex (> 6 conditions), while Patient Referenced Depth (Depth B) extracted 1479 rules with 69.6% complex rules, suggesting within patient network organization provides more interpretable classification criteria.

Systematic extraction of decision rules from the first 20 trees yielded 2,757 rules for Population Referenced Depth and 1,479 for Patient Referenced Depth (Figure 3). Rule complexity distributions showed Population Referenced Depth generated more complex rules (median 12 conditions) compared to Patient Referenced Depth (median 8 conditions), consistent with Population Referenced Depth’s more distributed variance structure. Simple rules (1 to 3 conditions) comprised roughly 1.6% of Population Referenced Depth rules and 2.4% of Patient Referenced Depth rules, offering readily interpretable biomarker signatures.

Example simple rules from Patient Referenced Depth included: “If JAK-STAT ↔ p53 depth ≤ 0.153 and Estrogen ↔ Hypoxia depth ≤ 0.319, predict Disease (LUAD)” and “If Hypoxia ↔ TGF*β* depth ≤ 0.125 and EGFR ↔ p53 depth > 0.139, predict Control (LUSC).” These rules suggest that combinations of reduced JAK-STAT ↔ p53 typicality with altered Estrogen ↔ Hypoxia coordination characterize LUAD, while LUSC shows distinct p53 and TGF*β* interaction patterns.

Top scoring pairs analysis identified 19 pairwise depth comparisons with AUC > 0.60 for Population Referenced Depth and 15 for Patient Referenced Depth. The highest performing Patient Referenced Depth pair compared Hypoxia ↔ JAK-STAT with Estrogen ↔ Hypoxia (AUC = 0.606), suggesting relative rather than absolute depth values carry discriminative information. Many top pairs involved p53 interactions for Population Referenced Depth and JAK-STAT or Hypoxia interactions for Patient Referenced Depth, indicating coordinated shifts in multiple pathway interaction typicalities distinguish subtypes.

### 2.5 Differential Expression Identifies Key Regulatory Pathways

**Figure 4.**
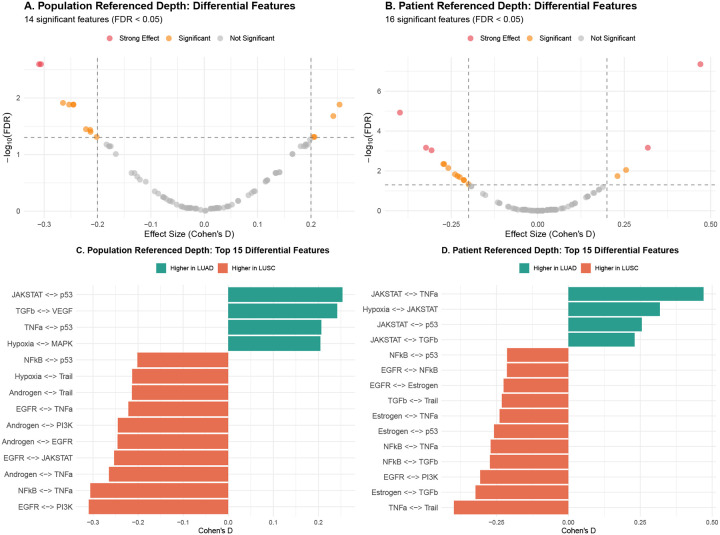
Differential expression analysis of pathway interaction features between LUAD and LUSC. (A,B) Volcano plots display effect sizes (Cohen’s D) versus statistical significance for Population Referenced Depth (14 significant features) and Patient Referenced Depth (16 significant features). Features with *FDR* < 0.05 are highlighted, with strong effects (|*D*| > 0.3) shown in red. (C,D) Top 15 differential features ranked by absolute effect size reveal distinct pathway disruption patterns. Population Referenced Depth emphasizes EGFR, PI3K, and NFkB interactions, while Patient Referenced Depth identifies TNFa, Estrogen, and JAKSTAT pathway pairs as most discriminative.

From Figure 4 we understand that for Patient Referenced Depth, JAK-STAT ↔ TNF*α* showed the strongest differential signal (Cohen’s D = 0.470, FDR < 0.001), with significantly higher depth in LUAD, indicating this interaction is more typical within LUAD patient networks. TNF*α* ↔ Trail showed strong negative regulation (Cohen’s D = −0.398, FDR < 0.001), being more typical in LUSC networks.

In Figure 4, panels A and B show volcano plots summarizing effect size (measured by Cohen’s D) and statistical significance (measured by false discovery rate) for each pathway interaction feature. Points highlighted as significant exceed an FDR threshold of 0.05, with stronger effects corresponding to larger absolute effect sizes. For Population Referenced Depth, a moderate number of features show statistically significant differences, with effect sizes distributed symmetrically across positive and negative values, indicating balanced pathway interaction shifts between the two subtypes. In contrast, Patient Referenced Depth displays a slightly larger set of significant features and a wider range of effect sizes, suggesting stronger subtype specific contrasts in within patient pathway organization.

Panels C and D summarize the top fifteen differential features ranked by absolute effect size for Population Referenced Depth and Patient Referenced Depth respectively. For Population Referenced Depth, features elevated in LUAD are enriched for interactions involving JAK-STAT, TGF*β*, TNF*α*, Hypoxia, and p53 signaling, while features elevated in LUSC predominantly involve EGFR, androgen related pathways, and NF*κ*B interactions. This pattern reflects systematic population level differences in pathway interaction structure between subtypes. For Patient Referenced Depth, the most differential features highlight coordinated shifts involving JAK-STAT and Hypoxia interactions in LUAD, contrasted with increased TNF*α*, Estrogen, EGFR, and NF*κ*B interactions in LUSC. Together, these results show that Population Referenced Depth captures broad population scale differences in pathway interactions, whereas Patient Referenced Depth emphasizes subtype specific reorganization of pathway relationships within individual patients.

Estrogen ↔ TGF*β* interactions also showed significant downregulation in LUAD (Cohen’s D = −0.323, FDR < 0.001), while Hypoxia ↔ JAK-STAT coordination was elevated (Cohen’s D = 0.318, FDR < 0.001). EGFR ↔ PI3K interactions, which are central to both subtypes’ biology, showed reduced typicality in LUAD (Cohen’s D = −0.307, FDR < 0.001). For Population Referenced Depth, p53 pathway interactions (EGFR ↔ p53, TGF*β* ↔ p53, TNF*α* ↔ p53, JAK-STAT ↔ p53) dominated differential signals, consistent with higher TP53 mutation frequency in LUSC.

Volcano plot analysis revealed 16 significantly differential features for Patient Referenced Depth (FDR < 0.05) versus 14 for Population Referenced Depth, though no features achieved large effect sizes (Cohen’s D > 0.8) in either model. This suggests that subtype discrimination emerges from coordinated patterns across multiple moderate effect features rather than individual high impact biomarkers.

### 2.6 Feature Co-occurrence Networks Identify Hub Interactions

To identify interdependent pathway interactions used by the classification models, we constructed feature co-occurrence networks from the random forest decision rules. For each depth representation (Population Referenced Depth and Patient Referenced Depth), we extracted decision rules from the top 20 trees in the trained random forest models. For each extracted rule, we identified all pathway features that appeared together in the decision path. When a rule contained features A, B, and C, we recorded pairwise co-occurrences for (A,B), (A,C), and (B,C). The total co-occurrence count for each pair was aggregated across all rules. We constructed network graphs where nodes represent individual pathway interactions and edges represent co-occurrence relationships. Edge width and transparency were scaled proportionally to co-occurrence frequency, with more frequently paired pathways shown with thicker, more opaque connections. Node size was determined by degree centrality, reflecting the number of distinct pathways with which each feature co-occurs. Node color intensity represented betweenness centrality, quantifying each pathway’s importance as a bridge connecting different network modules.

**Figure 5.**
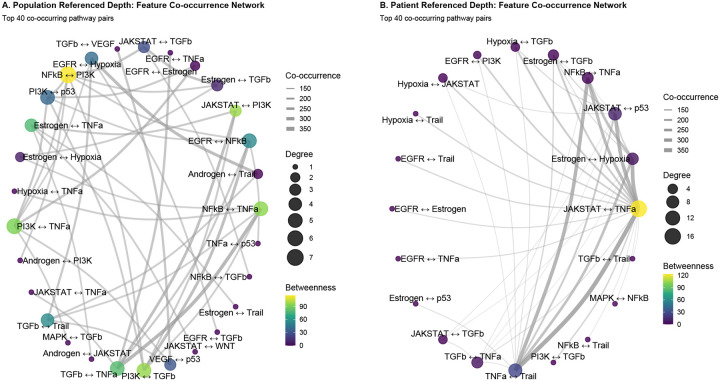
Co-occurrence network analysis of pathway pairs in random forest decision rules. Networks display the top 40 most frequently co-occurring feature pairs extracted from decision trees. Node size represents degree centrality, node color indicates betweenness centrality, and edge width corresponds to co-occurrence frequency. (A) Population Referenced Depth network shows VEGF and p53 as central hubs with extensive connectivity. (B) Patient Referenced Depth network reveals JAKSTAT and TNFa as key coordinators with high betweenness, suggesting their role as bridges between pathway modules. Both networks exhibit distinct topological structures reflecting different biological coordination patterns captured by each depth reference frame.

We generated networks (Figure 5) using the top 40 most frequently co-occurring pathway pairs. Networks were visualized using a circular layout to enhance readability and comparison between Population Referenced Depth and Patient Referenced Depth representations. This layout distributes nodes evenly around a circle while preserving the edge structure, making it easier to identify dense connectivity patterns and central hub features. These networks reveal functional modules of pathway interactions that the random forest models use jointly for LUAD versus LUSC classification. Densely connected clusters indicate groups of pathways that work together in the classification decision. Pathways with high betweenness centrality serve as critical connectors between different biological processes, suggesting they may represent key regulatory nodes that distinguish the two lung cancer subtypes.

In panel A (Figure 5), the Population Referenced Depth network shows a distributed connectivity structure with multiple highly connected hubs spanning EGFR, JAK-STAT, NF*κ*B, TNF*α*, p53, Hypoxia, and hormone related pathways. The presence of several high degree and high betweenness nodes indicates that population level pathway organization is characterized by a broadly interconnected architecture in which multiple signaling modules jointly contribute to classification. In panel B (Figure 5), the Patient Referenced Depth network shows a more centralized structure dominated by a small number of highly connected nodes, most prominently JAK-STAT and TNF*α* interactions. Many peripheral nodes display lower degree and reduced betweenness, indicating that within patient pathway organization concentrates around a narrower set of coordinating interactions. These contrasting network topologies show that Population Referenced Depth captures widespread population scale co-occurrence patterns across many pathways, whereas Patient Referenced Depth highlights focused within patient coordination driven by a small number of dominant signaling interactions.

To understand how features coordinate in classification decisions, we built co-occurrence networks from extracted rules (Figure 5). For Population Referenced Depth, the network had 68 features with 440 co-occurrence relationships. Hub features with highest degree centrality included PI3K ↔ Trail (degree 31), Androgen ↔ Trail (degree 31), Androgen ↔ JAK-STAT (degree 28), and Androgen ↔ Estrogen (degree 26). PI3K ↔ Trail showed exceptional total co-occurrence (194 across all pairs), suggesting central importance to classification logic.

For Patient Referenced Depth, JAK-STAT ↔ TNF*α* emerged as the dominant hub (degree 69, total co-occurrence 6,010), followed by TNF*α* ↔ Trail (degree 67, co-occurrences 3,907) and NF*κ*B ↔ TNF*α* (degree 70, co-occurrences 3,682). The most frequently co-occurring pair was NF*κ*B ↔ TNF*α* with TGF*β* ↔ TNF*α* (308 co-occurrences), indicating coordinated inflammatory signaling assessment in Patient Referenced Depth classification.

The co-occurrence frequency distributions revealed contrasting patterns. Population Referenced Depth showed relatively uniform co-occurrence (median 46) with a maximum of 308, suggesting many features contribute similarly. Patient Referenced Depth showed a highly right skewed distribution (median 9, maximum 364), indicating concentration of discriminative power in a smaller set of highly coordinated inflammatory and immune signaling interactions.

### 2.7 Random Forest Feature Importance Highlights Distinct Pathway Networks

To identify the most informative pathway interactions for LUAD versus LUSC classification, we extracted feature importance scores from the trained random forest models using permutation importance. This approach measures the decrease in model performance when each feature’s values are randomly permuted, quantifying each pathway’s contribution to predictive accuracy. For both Population Referenced Depth and Patient Referenced Depth representations, we ranked all pathway features by their permutation importance scores and identified the top 30 features. To assess consistency between the two depth approaches, we compared the top 15 features from each method and quantified their overlap. This analysis reveals whether population-level depth patterns (Population Referenced Depth) and within-patient depth patterns (Patient Referenced Depth) identify similar or distinct pathway signatures. We integrated random forest feature importance with differential expression analysis to identify pathway interactions that are both statistically significant and predictively powerful. For each feature, we combined two complementary rankings: (1) rank by absolute Cohen’s D effect size from differential expression testing, and (2) rank by random forest permutation importance. The combined score was calculated as Combined ${\text{Score}}_{i}=\frac{1}{2}\left(\frac{1}{{\text{Rank}}_{\text{Diff},i}}+\frac{1}{{\text{Rank}}_{\text{Imp},i}}\right)$ where higher scores indicate features that rank highly by both criteria. This multi-method integration prioritizes pathway interactions that show strong biological differences between cancer subtypes (high Cohen’s D) while also contributing substantially to classification performance (high RF importance). We visualized the relationship between differential expression and predictive importance through scatter plots, with point size scaled by the combined score. Features with FDR-adjusted p-values below 0.05 were colored to highlight statistically significant pathway interactions. This integrative approach identifies robust biomarker candidates that satisfy both statistical significance and predictive utility criteria, reducing the likelihood of selecting spurious features that excel in only one dimension.

**Figure 6.**
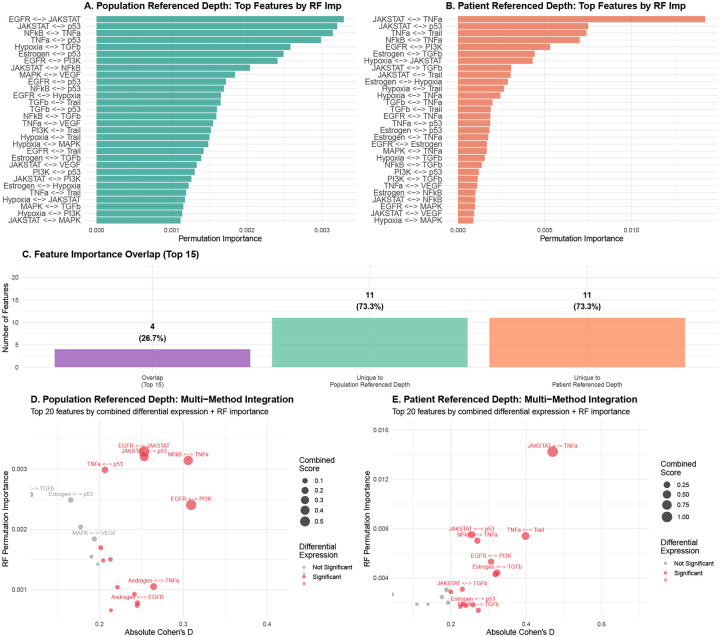
Feature importance analysis and multi-method integration. (A,B) Top 30 features ranked by random forest permutation importance show partially overlapping but distinct priority sets for Population Referenced Depth and Patient Referenced Depth. (C) Venn diagram reveals 73.3% unique features in each model’s top 15, indicating complementary discriminative patterns. (D,E) Multi-method integration plots combine differential expression (Cohen’s D) with machine learning importance, bubble size represents combined score. Top ranked features balance statistical significance with predictive power, with Population Referenced Depth emphasizing EGFR and JAKSTAT pathways, while Patient Referenced Depth prioritizes TNFa and TGFb coordination patterns.

Figure 6 summarizes feature importance and cross method integration for the population level Population Referenced Depth representation and the within patient Patient Referenced Depth representation. Panels A and B display the top thirty pathway interaction features ranked by random forest permutation importance for Population Referenced Depth and Patient Referenced Depth respectively. For Population Referenced Depth, importance scores are more evenly distributed across a broad set of pathway interactions, with prominent contributions from EGFR, JAK-STAT, NF*κ*B, TNF*α*, p53, Hypoxia, and hormone related signaling pathways. This pattern indicates that population level classification relies on multiple moderately influential interactions rather than a single dominant feature. In contrast, Patient Referenced Depth shows a more concentrated importance profile, with JAK-STAT and TNF*α* interactions contributing disproportionately to classification performance, suggesting that within patient discrimination is driven by a smaller subset of highly influential pathway interactions.

Panel C quantifies overlap among the top fifteen important features for the two depth representations. Only four features are shared between Population Referenced Depth and Patient Referenced Depth, while the majority of highly ranked features are unique to each representation. This limited overlap shows that population level and within patient depth capture complementary but distinct aspects of pathway interaction structure.

Panels D and E integrate differential expression effect size and random forest importance to identify features supported by multiple lines of evidence. For Population Referenced Depth, several features achieve simultaneously large absolute Cohen’s D values and high permutation importance, indicating robust population scale discriminative power. These features include pathway interactions involving p53, JAK-STAT, TNF*α*, and EGFR signaling. For Patient Referenced Depth, fewer features occupy the extreme upper right region of the joint importance and effect size space, reflecting more moderate but coordinated contributions across multiple interactions. Together, these results show that Population Referenced Depth emphasizes globally consistent pathway interaction differences across the cohort, whereas Patient Referenced Depth emphasizes within patient relative pathway organization driven by a smaller set of dominant signaling interactions.

Permutation importance analysis revealed divergent feature prioritization between models (Figure 6 D and E). For Population Referenced Depth, top features included EGFR ↔ JAK-STAT (importance 0.00329), JAK-STAT ↔ p53 (0.00320), NF*κ*B ↔ TNF*α* (0.00314), TNF*α* ↔ p53 (0.00299), and Hypoxia ↔ TGF*β* (0.00258). This prioritization highlights EGFR signaling coordination, p53 network interactions, and inflammatory pathway cross talk.

Patient Referenced Depth showed markedly different priorities: JAK-STAT ↔ TNF*α* (0.01425, over 4 fold higher than any Population Referenced Depth feature), JAK-STAT ↔ p53 (0.00751), TNF*α* ↔ Trail (0.00740), NF*κ*B ↔ TNF*α* (0.00701), and EGFR ↔ PI3K (0.00531). The dramatically elevated importance of JAK-STAT ↔ TNF*α* in Patient Referenced Depth, combined with its strong hub centrality, establishes this interaction as the primary discriminative feature when assessing within patient network organization.

Feature importance overlap analysis revealed 18 features appearing in the top 30 for both models, with 12 unique to each approach. This moderate overlap (60%) suggests partially distinct disease specific biological information capture, explaining why Patient Referenced Depth’s superior performance cannot be simply predicted from Population Referenced Depth results.

## 3 Discussion

### 3.1 Statistical Framework and Main Findings

This study establishes a computational framework integrating functional data analysis with network-based feature engineering for lung cancer biomarker discovery. We transform high-dimensional pathway interaction networks into interpretable features through depth statistics, comparing within-patient and population-level reference frames.

Random forest classification revealed distinct performance profiles for the two depth approaches (Table 1, Figure 2). Patient Referenced Depth achieved test AUC of 0.768 with accuracy of 70.8%, while Population Referenced Depth yielded comparable AUC of 0.751 with accuracy of 69.1%. Critically, the models exhibited contrasting sensitivity-specificity trade-offs: Patient Referenced Depth favored specificity (74.5%) over sensitivity (67.5%), whereas Population Referenced Depth prioritized sensitivity (75.2%) at the expense of specificity (62.4%).

The contrasting profiles suggest these approaches capture fundamentally different biological information. Patient Referenced Depth achieves superior specificity and AUC through within-patient network organization, providing more precise identification of coordinated signaling patterns. This advantage mirrors results from individual-level pathway methods (TPAC^31^, VAM^21^, N-of-1-pathways^22^), which consistently outperform population-based approaches. Out of bag estimates closely matched test performance, confirming stable generalization. We focus subsequent analyses on Patient Referenced Depth given its superior discriminative properties and precision medicine implications.

Systematic integration of feature selection methods identifies biologically coherent signatures. JAK-STAT ↔ TNF*α* emerges as the dominant discriminative feature: highest random forest importance (0.01425), largest effect size (Cohen’s D = 0.470), and maximum network centrality (degree 69). This convergence confirms inflammatory signaling coordination drives subtype differentiation^32,33^.

Feature co-occurrence networks reveal fundamentally different organizational principles. Patient Referenced Depth exhibits extreme hub concentration around inflammatory interactions (JAK-STAT ↔ TNF*α*, NF*κ*B ↔ TNF*α*), indicating classification depends on few highly coordinated features. Population Referenced Depth shows distributed topology with uniform degree distribution, relying on aggregate assessment across many weakly connected features. Concentrated discriminative power in parsimonious feature sets provides more stable decision boundaries^13,34^.

### 3.2 Functional Depth as Computational Strategy for Biomarker Discovery

The functional depth framework addresses computational challenges in high-dimensional omics data. Traditional differential expression identifies shifted marginal distributions but misses complex multivariate patterns involving coordinated pathway dysregulation^28,35^. Depth measures quantify observation centrality within multivariate distributions, capturing distributional properties beyond location shifts.

The Fraiman-Muniz depth statistic^35^ integrates pointwise depths across functional domains, transforming graph-structured data into scalar features while preserving global network organization. Subsampling-based depth estimation enhances robustness and provides stable estimates with moderate sample sizes.

Patient Referenced Depth uses each patient’s own pathway network as the reference frame, measuring deviations from their individual baseline rather than comparing to population averages. This captures patient-specific coordination patterns that may be masked when using population-level comparisons^36^. Clinical evidence supports this individualized approach. The I-PREDICT trial demonstrated that matching therapies to individual molecular profiles improved objective response rates and extended progression-free survival^19^. Similarly, a pan-cancer Molecular Tumor Board analysis found that high therapy-biomarker concordance at the individual level predicted longer survival, while no population-level biomarker was independently predictive^20^.

Population Referenced Depth’s severe imbalance (high specificity, poor sensitivity) indicates substantial heterogeneity in population-level pathway organization within LUSC, consistent with evidence that LUSC exhibits greater molecular diversity than LUAD^37,38^. Patient Referenced Depth’s balanced performance indicates within-patient network patterns provide consistent discrimination across both subtypes.

### 3.3 Biological Interpretation: Depth Patterns Reveal Three Mechanistic Frameworks

Depth-based network analysis identifies three coordinated pathway frameworks distinguishing LUAD from LUSC: immune-inflammatory coordination, mutation-driven rewiring, and microenvironment-driven programming.

#### 3.3.1 Immune-Inflammatory Coordination

JAK-STAT and TNF*α* pathways show the strongest differential coordination. LUAD exhibits tight JAK-STAT ↔ TNF*α* coupling (Cohen’s D = 0.318, *FDR* < 0.001), driven by SPP1^+^ macrophages that co-localize with cancer-associated fibroblasts in spatially organized inflammatory niches^39^. These macrophages coordinate hypoxia response through direct STAT3-HIF-1*α* interaction, where STAT3 stabilizes HIF-1*α* and regulates approximately 30% of hypoxia-induced genes^9^. Spatial transcriptomics confirms TP53-mutant LUAD develops multicellular ecosystems where SPP1^+^ macrophages, fibroblasts, and malignant cells coordinately regulate metabolic and immune suppressive programs in hypoxic regions^7^.

LUSC shows fundamentally different inflammatory architecture. NF*κ*B ↔ TNF*α* coordination dominates, operating through TNFR1 signaling that drives dedifferentiation programs^11^. Near-universal TP53 mutations (82% versus 47% in LUAD) amplify this effect by disrupting NF-*κ*B regulation^3^. The TNF*α* ↔ TRAIL interaction (Cohen’s D = −0.398) reflects coordinated death receptor signaling where LUSC cells resist TRAIL-induced apoptosis through constitutive NF-*κ*B activation despite upregulated death receptors in necrotic tumor cores.

#### 3.3.2 Mutation-Driven Pathway Rewiring

p53 pathway interactions dominate Population Referenced Depth features (EGFR ↔ p53, TGF*β* ↔ p53, TNF*α* ↔ p53, JAK-STAT ↔ p53), but depth analysis reveals network consequences beyond mutation frequency. TP53 mutations trigger more heterogeneous phenotypic changes in LUAD than LUSC: multi-omic analysis of 992 NSCLC patients demonstrated deep learning achieved AUC 0.84 for predicting TP53 mutation from histology in LUAD but failed in LUSC^40^. In LUAD, TP53 mutations occur in approximately half of tumors as later clonal events, triggering ecosystem reorganization captured by Population Referenced Depth. In LUSC, near-universal early TP53 mutation creates homogeneous baseline better discriminated by Patient Referenced Depth.

EGFR pathway coordination patterns reflect distinct activation modes. LUAD shows reduced EGFR ↔ PI3K typicality (Cohen’s D = −0.307) where activating mutations (10–15% of cases) enable ligand-independent signaling, uncoupling EGFR from normal PI3K regulation. LUSC maintains tight EGFR-PI3K coordination despite EGFR amplification (20% of cases) through ligand-dependent signaling requiring autocrine loops^3,37^. Recently characterized EGFR-TNFR1 physical interaction explains LUSC therapeutic resistance: EGFR phosphorylates TNFR1 death domain, suppressing NF-*κ*B activation, such that EGFR inhibition paradoxically enables compensatory survival signaling^41^.

#### 3.3.3 Microenvironment-Driven Programming

Hypoxia and hormone pathway coordination reflects anatomical constraints. LUAD arises peripherally in well-vascularized regions experiencing fluctuating hypoxia with intermittent reoxygenation. This creates selective pressure for coordinated HIF-1*α*/STAT3 signaling maintaining chronic inflammation and angiogenesis^9^. VEGF ↔ WNT interaction emerges as a dominant Patient Referenced Depth principal component, reflecting integration of angiogenic and developmental signaling where WNT activation occurs in 30–50% of LUAD through various alterations co-occurring with EGFR mutations^37^.

LUSC develops centrally near airways with chronic stable hypoxia and large necrotic cores. Central location constrains neovascularization, with LUSC relying on existing bronchial vasculature rather than tumor-induced angiogenesis. This explains reduced VEGF coordination and differential anti-angiogenic therapy responses.

Hormone pathway enrichment in LUSC (Androgen ↔ TRAIL, EGFR ↔ Estrogen, Estrogen ↔ TGF*β*) is notable given 75% male predominance. Androgen receptor inhibits effector and stem cell properties of male tumor-infiltrating CD8^+^ T cells, creating male-biased terminal exhaustion, with castration plus anti-PD-L1 synergistically restricting tumor growth^42^. Androgen ↔ TRAIL coordination may reflect hormone-mediated modulation of death receptor sensitivity creating sex-specific apoptosis resistance.

#### 3.3.4 Pathway Interaction Patterns and Molecular Subtype Heterogeneity

Our pathway interaction signatures align with known KRAS-mutant and ALK-fusion biology, though definitive attribution requires matched genomic data. The spatial organization patterns captured through pathway proximity networks suggest how these molecular alterations restructure signaling architectures. Reduced EGFR ↔ PI3K typicality in LUAD (Cohen’s D = −0.307) reflects spatial signaling reorganization characteristic of KRAS-mutant tumors. Constitutive RAS-GTP maintains MAPK activity independent of receptor regulation, spatially uncoupling EGFR from PI3K engagement. Spatial profiling studies demonstrate KRAS mutations disrupt growth factor receptor clustering and alter stromal positioning^43^, architectural features manifesting as reduced pathway proximity depths in our network analysis. JAK-STAT ↔ TNF*α* dominance (importance 0.01425, Cohen’s D = 0.470) likely captures KRAS-driven inflammatory niche organization. KRAS mutations activate NF-*κ*B signaling, creating spatially coordinated inflammatory ecosystems with SPP1^+^ macrophages and cancer-associated fibroblasts^44^. Single-cell spatial mapping confirms KRAS-mutant tumors establish inflammatory architectures with defined cytokine signaling zones^45^, directly supporting our observed coordination patterns. ALK fusions activate overlapping pathways through distinct spatial mechanisms. Constitutive ALK signaling phosphorylates STAT3 independent of cytokine receptors, creating JAK-STAT activation with different spatial distributions than KRAS inflammatory niches. ALK-fusion tumors exhibit metabolically active hypoxic cores with peripheral immune exclusion^46^. Elevated Hypoxia ↔ JAK-STAT coordination in LUAD (Cohen’s D = 0.318) aligns with this biology, where STAT3 activation occurs in hypoxic domains lacking KRAS inflammatory infiltrates. MAPK serves as a convergent downstream effector but shows distinct spatial organization. KRAS-mutant tumors exhibit diffuse MAPK activation across tumor and stromal compartments, while ALK-fusion tumors show localized MAPK activity in malignant populations with defined spatial boundaries.

ROS1 fusions, present in 1–2% of LUAD, provide a further mechanism through which JAK-STAT and PI3K pathway interactions become reorganized relative to EGFR signaling. In LUAD, ROS1 fusion proteins signal constitutively through SHP2, activating JAK/STAT3, PI3K/AKT/mTOR, and RAS/MEK/ERK cascades in the absence of ligand-mediated receptor engagement^47,48^. Transcriptomic profiling of ROS1-positive LUAD relative to ALK-positive tumors reveals upregulation of interleukin-17 signaling and nucleotide synthesis pathways^49^, suggesting a cytokine niche distinct from ALK-driven ecosystems. Crucially, ROS1-positive LUAD presents with low tumor mutational burden and an immune-cold microenvironment characterized by poor response to immune checkpoint inhibitors^49,50^, consistent with the reduced EGFR ↔ PI3K depth typicality we observe in LUAD relative to LUSC. In LUSC, receptor tyrosine kinase pathway coordination remains more prominent, and the ROS1 fusion-driven decoupling of EGFR from PI3K engagement in LUAD may partially explain the depth asymmetry captured by our patient-referenced features. The partner-dependent localization of ROS1 fusions further modulates which downstream branches are amplified: SLC34A2-ROS1 and CD74-ROS1 variants in LUAD associate with distinct PI3K/AKT/mTOR activation profiles at the single-nucleus level^51^, supporting the interpretation that ROS1 contributes heterogeneous but coherent depth signatures within our LUAD cohort.

METΔex14 mutations, occurring in 3–4% of LUAD, impair CBL-mediated receptor degradation by removing the Y1003 ubiquitin ligase binding site, leading to sustained surface accumulation of MET and prolonged HGF-dependent signaling^52,53^. In LUAD cell lines and TCGA samples, METΔex14 preferentially amplifies RAS-MAPK activation, with cell-line-specific co-activation of PI3K/AKT and STAT3 that depends on co-occurring genetic context^54^. This simultaneous engagement of MAPK, STAT3, and PI3K from a single receptor platform produces dense pathway co-activation that depth-based features are designed to detect. The immune microenvironment of METΔex14 LUAD is heterogeneous: mIF profiling of surgically resected LUAD shows exhausted CD8^+^TIM3^+^ and CD8^+^LAG3^+^ cells are spatially redistributed between tumor parenchyma and stroma depending on recurrence status, while immune checkpoint genes including CTLA4, PD-1, LAG3, and TIGIT are broadly upregulated^55,56^. This spatially reorganized immune architecture, driven in part by MET-mediated neutrophil recruitment and myeloid suppression, is consistent with the high hub centrality of NF*κ*B ↔ TNF*α* in Patient Referenced Depth co-occurrence networks, where coordinated inflammatory signaling reflects stromal-immune crosstalk. Stratified analysis integrating whole exome sequencing with spatial transcriptomics represents a critical extension. The mutual exclusivity of ROS1 fusions and METΔex14 with other LUAD drivers^57,58^ supports the use of pathway proximity networks as a driver-agnostic profiling strategy capable of detecting subtype-defining signaling architectures without prior molecular characterization.

#### 3.3.5 Therapeutic Implications

Five novel LUSC-enriched interactions (Androgen ↔ TRAIL, EGFR ↔ Estrogen, EGFR ↔ TNF*α*, Hypoxia ↔ TRAIL, TGF*β* ↔ TRAIL) lack mechanistic characterization in squamous lung cancer, while LUAD signatures are well-established. This asymmetry reveals critical knowledge gaps potentially explaining limited LUSC therapeutic options: no FDA-approved targeted therapies beyond general EGFR inhibitors with limited efficacy, versus multiple actionable LUAD mutations (EGFR, ALK, ROS1, KRAS G12C) that transformed adenocarcinoma treatment^2^.

Convergence of uncharacterized interactions on TRAIL signaling suggests underappreciated death receptor roles in LUSC. Central tumor location creates unique microenvironmental gradients where viable tumor regions show differential death receptor expression. TGF*β* modulates TRAIL sensitivity through death receptor expression and anti-apoptotic protein regulation^59^, suggesting coordinated resistance mechanisms warrant investigation. Combined EGFR inhibition with anti-TNF*α* agents may address compensatory NF-*κ*B activation^41^, while androgen receptor blockade with PD-L1 inhibitors warrants evaluation in male LUSC patients^42^.

### 3.4 Methodological Limitations and Future Directions

Several limitations merit consideration. Our analysis employs 14 PROGENY pathways representing major cancer signaling axes, which may not capture all relevant biological processes. Future work should incorporate additional pathway databases or extend to gene-level graphical models, with external validation on independent cohorts essential for assessing generalizability. Cross-platform validation across spatial transcriptomics, bulk RNA-seq, and single-cell RNA-seq would further strengthen translational potential. The correlational nature of our findings precludes causal inference, which could be addressed by integrating CRISPR perturbation screens, drug response data from patient-derived organoids, and longitudinal clinical outcomes. Existing organoid biobanks for LUAD and LUSC^60,61^ provide experimental platforms for such validation efforts. Additionally, our transcript-based analysis does not capture protein-level regulation and post-translational modifications that govern many signaling pathways. Complementary approaches using multiplex immunofluorescence (mIF) or spatial proteomics would validate our transcriptomic findings and enable direct assessment of pathway activity at the functional level, representing a natural direction for future studies.

We employed binary classification despite substantial molecular heterogeneity within subtypes. Extending functional depth to multi-class classification could reveal pathway interaction signatures distinguishing finer-grained molecular subtypes with distinct therapeutic vulnerabilities. Simplified expression panels targeting identified pathway interaction signatures could enable clinical implementation. Reverse engineering compact gene expression panels recapitulating pathway network depth features using L1-regularized regression could produce parsimonious assays.

### 3.5 Conclusions

Functional depth analysis of pathway interaction networks provides a statistically principled, biologically interpretable framework for biomarker discovery in lung cancer molecular subtypes. Within-patient network depth achieves 71.1% classification accuracy while revealing systems-level organization. The superior performance of Patient Referenced Depth demonstrates that internal signaling architecture more robustly captures subtype-specific molecular features than population comparisons, with direct support from clinical N-of-1 trials showing therapies matched to individual molecular profiles significantly improve outcomes.

The pathway interaction signatures exhibit remarkable biological coherence with TCGA molecular landscapes and spatial profiling studies. JAK-STAT ↔ TNF*α* emerges as dominant LUAD hub, TNF*α* ↔ Trail distinguishes LUSC through coordinated death receptor programs, p53 network interactions capture subtype-specific consequences of differential TP53 mutation frequencies, and hypoxia-related coordination patterns reflect anatomical tumor locations. Five novel LUSC-enriched interactions represent high-priority research opportunities, revealing critical knowledge gaps that may explain limited therapeutic options. This work establishes functional depth as a valuable computational tool for precision oncology, providing a generalizable framework for extracting interpretable, molecularly validated biomarkers from high-dimensional biological networks.

## 4 Methods

### 4.1 Data Acquisition and Preprocessing

We obtained spatial transcriptomics data for lung cancer patients from a publicly available dataset resulting from the DeepSpot^62^. DeepSpot is a deep learning method for predicting spatially resolved transcriptomics from H&E data. Specifically, DeepSpot leverages pathology foundation models and tissue context across multiple scales to perform state-of-the-art prediction of spatial transcriptomics. They applied and validated DeepSpot on H&E slides from The Cancer Genome Atlas (TCGA) database, including those of lung cancer (both LUAD and LUSC subtypes). This dataset had 996 patient samples (525 LUAD, 471 LUSC). Each sample consisted of spatially resolved gene expression measurements. Quality control removed low quality spots and samples with insufficient coverage.

### 4.2 Pathway Activity Inference

We used PROGENY^29^ to infer pathway activities from spatial transcriptomics data. PROGENY uses a linear model relating gene expression to pathway activity: **E** = **W · A** + *ε* where **E** is gene expression, **W** is the weight matrix encoding gene responsiveness to pathways, **A** is pathway activity, and *ε* is noise. We focused on 14 cancer related pathways: Androgen, Estrogen, Hypoxia, JAK-STAT, MAPK, NF*κ*B, p53, PI3K, TGF*β*, TNF*α*, Trail, VEGF, WNT, and EGFR. For each sample, we computed activities using the PROGENY R package with default parameters (top 500 responsive genes per pathway). This resulted in a vector of pathway activity scores for each spot in the spatial transcriptomics data. To determine the most enriched pathway per spot, we assigned each spot to the pathway with the highest activity score.

### 4.3 Construction of Pathway Interaction Graphs

For each patient, we built a pathway interaction graph *G* = (*V,E*) where nodes *V* correspond to the 14 pathways and edges *E* capture spatial proximity interaction strength. We quantified interaction strength between pathways *i* and *j* using partial correlation across spatial locations. When a pathway projection was absent from a sample, we set all incoming and outgoing edges to zero. For samples with pathway presence, edges encoded the G-cross spatial interaction between pathway pairs according to the following equation^30^:

(1) $$G_{i,j}(R)=\frac{1}{N}\sum_{r=0}^{R}\sum_{k=1}^{N}I\left\{d_{i[k],j}<r\right\}$$

where *i* and *j* indicate two pathway types, *N* denotes the total count of instances for pathway type *i* in the sample, *d*_*i*[*k*],*j*_ represents the nearest neighbor distance, and *R* specifies the computation radius of 150 microns, corresponding to about 3 spots. Because G-cross is asymmetric, we used directed edges between nodes. We then derived an undirected adjacency matrix by keeping only reciprocal connections, meaning edges that existed in both directions in the original directed graph. Formally, this is expressed as *Ad j* = (*A* > 0∧*A*^*T*^ > 0), guaranteeing that an undirected edge between nodes *i* and *j* exists only when both *A*_*ij*_ and *A*_*ji*_ are nonzero. This procedure captures mutual relationships while removing one way links, producing a symmetric representation suitable for analyzing bidirectional or co-occurrence network structures. while G-cross was used here, any other preferred function of proximity can be used as well.

By design, certain nodes emerged as zeros in our construction, requiring imputation of missing edges. These missing nodes, appearing as all zero rows and columns, likely resulted from technical limitations in data acquisition rather than genuine absence of connectivity. We used soft imputation, a low rank matrix completion method, to reconstruct missing edge weights by leveraging observed connectivity patterns throughout the network. The algorithm assumes the adjacency matrix has an underlying low rank structure, allowing prediction of plausible edge weights for missing nodes through latent relationships among observed nodes. After imputation, we applied post processing steps including diagonal normalization, non-negativity constraints, and symmetrization to guarantee the resulting adjacency matrix satisfied the necessary properties of an undirected weighted graph. Importantly, our approach imputed entries only for completely unobserved nodes while maintaining all structural zeros in partially observed nodes, thus avoiding spurious edge introduction. This imputation framework enabled retention of samples that would otherwise be discarded due to incomplete node level data, increasing statistical power for downstream network analyses.

To ensure robust estimation, we applied soft imputation using the softImpute algorithm^63^ with regularization parameter λ = 0.1 to impute missing edges in a graph. Because adjacency matrices cannot be directly used, we converted them into covariance matrices. To estimate the overall covariance pattern characterizing the graph, we used an approach based on additive decomposition^64^. A function *g*(*x*) of a variable *x* can be written in additive form as (*g*(*x*) = *c*+∑_*i*_ *g*_*i*_(*x*_*i*_))^65^. We estimated the covariance through additive decomposition of additive functions of Gaussian processes over the domain^66^. We then extracted partial correlations from the inverse covariance matrix to obtain the binary graph. From each adjacency matrix, we extracted upper triangular elements, yielding 91 edge weights per patient.

### 4.4 Train-Test Split and Depth Computation

To prevent data leakage, we performed train-test splitting before depth computation. We split the dataset 70–30 using stratified sampling to maintain class proportions (training: 698 samples, 330 LUSC, 368 LUAD; test: 298 samples, 141 LUSC, 157 LUAD).

#### 4.4.1 Fraiman-Muniz Depth with Subsampling

We employed the Fraiman-Muniz depth statistic^25^ to quantify centrality of pathway interaction profiles. For patient *i* with edge weight vector **x**_*i*_ = (*x*_*i*,1_,…,*x*_*i*,91_) over the discrete domain 𝒯 = {1,2,…,91}, the FM depth is:

(2) $$D_{FM}\left(x_{i}\right)=\frac{1}{91}\sum_{j=1}^{91}D_{u}\left(x_{i,j},F_{j}\right)$$

where *D*_*u*_(*x*_*i,j*_,*F*_*j*_) denotes the univariate depth of edge *j*’s weight in patient *i* with respect to the marginal distribution *F*_*j*_ of edge *j* across the reference population. This formulation treats the 91 edge weights as discretely sampled evaluations of a patient-specific pathway interaction function, enabling application of functional data depth methods to graph-structured biological data.

To ensure stability, we implemented a subsampling approach. For each depth computation, we performed *B* = 300 bootstrap iterations, where each iteration randomly sampled 80% of the reference population without replacement. The trimming parameter was set to 0.15 to reduce sensitivity to outliers. For each observation, we computed depth values across all *B* iterations and took the median as the final depth score, providing robust estimates less sensitive to sampling variation.

#### 4.4.2 Population Referenced Depth: Across-Patient Depth

For Population Referenced Depth, we measured how central each patient’s edge connectivity is relative to the population. For each edge *j* and patient *i*, we computed patient referenced depth treating patient *i*’s connectivity value for edge *j* as a functional observation and comparing it against the distribution of connectivity values across all training patients for that same edge.

Critically, for test patients, we employed a specialized scoring function to prevent data leakage. Rather than computing depth using the combined train-test distribution, we computed depth for test observations using only the training data as the reference distribution. Specifically, for each edge *j*, we combined training and test connectivity values into a single vector, then performed *B* = 300 subsampling iterations where each iteration sampled 80% of training indices only. We computed FM depth for all observations relative to each training subsample, then extracted depth values corresponding to test observations and aggregated via median across iterations. This ensured test patients were scored relative to patterns learned from training data alone, maintaining proper separation between training and validation phases.

#### 4.4.3 Patient Referenced Depth: Within-Patient Depth

For Patient Referenced Depth, we measured how central each edge is within an individual patient’s connectivity profile. For each patient *i* and edge *j*, we computed Patient Referenced Depth by treating edge *j*’s connectivity as a functional observation and comparing it against the distribution of all other edges for that same patient.

Unlike population referenced depth, patient referenced depth computation does not require special handling for test data because each patient’s depth profile is independent of other patients. Both training and test patients were scored using their own internal edge distributions. For patient *i*, we performed *B* = 300 subsampling iterations, where each iteration sampled 80% of that patient’s edges, computing how central each edge *j* is relative to the sampled edge distribution. The final within-patient depth was obtained by taking the median across all iterations.

This dual depth representation captures both population-level patterns (Population Referenced Depth) and patient-specific connectivity architecture (Patient Referenced Depth), providing complementary information for disease classification.

### 4.5 Justification for Functional Data Representation

Our application of functional depth statistics to graph-structured data requires explicit justification, as pathway interaction networks are inherently discrete rather than continuous functional observations. We adopt a framework where the 91 edge weights constitute evaluations of an underlying latent function over a discrete index domain. Formally, we define the domain 𝒯 = {1,2,…,91} as the discrete index set corresponding to the 91 unique pathway pairs (edges) in the complete graph. For patient *i*, the edge weight vector **x**_*i*_ = (*x*_*i*,1_,*x*_*i*,2_,…,*x*_*i*,91_) represents evaluations of a patient-specific function $f_{i}:𝒯\to R^{+}$ that characterizes their pathway interaction profile. Each edge index *j* ∈ 𝒯 maps to a specific pathway pair (e.g., *j* = 1 corresponds to Androgen ↔ Estrogen, *j* = 2 to Androgen ↔ Hypoxia, etc.). This discrete functional representation is justified on three grounds. First, functional data analysis methods, including depth statistics, extend naturally to discretely sampled functions^67^. The Fraiman-Muniz depth integrates univariate depths across the domain via: $D_{FM}\left(f_{i}\right)=\frac{1}{\left|𝒯\right|}\sum_{j\in 𝒯}D_{u}\left(x_{i,j},F_{j}\right)$ where the integral in the continuous formulation reduces to a discrete sum over the 91 edges, *D*_*u*_(*x*_*i,j*_,*F*_*j*_) is the univariate depth of patient *i*’s value for edge *j* relative to the marginal distribution *F*_*j*_ (for Population Referenced Depth), and |𝒯| = 91 normalizes the sum. This formulation treats each patient’s pathway interaction profile as a vector-valued observation while leveraging the depth framework’s ability to capture centrality in high-dimensional spaces. Second, if we focus on the biological interpretation, viewing edge weights as a function over pathway pair indices reflects the biological principle that pathway interactions form coordinated regulatory programs rather than independent entities. The functional perspective captures global network organization patterns which translates to a patient’s complete signaling architecture rather than treating individual edges as isolated measurements. This aligns with systems biology perspectives where cellular phenotypes emerge from coordinated pathway cross-talk^12^. And third, being the computational advantages i.e., functional depth provides a principled dimensionality reduction from 91-dimensional vectors to scalar centrality scores while preserving multivariate structure. Unlike univariate approaches that analyze edges independently, or simple averaging that discards information, functional depth quantifies how typical a patient’s entire pathway interaction profile is relative to a reference distribution, naturally handling the high-dimensional, correlated structure of biological networks.

For Population Referenced Depth (across-patient), the reference distribution *F*_*j*_ for each edge *j* is the marginal distribution of that edge’s weights across the patient population. For Patient Referenced Depth (within-patient), we redefine the domain as 𝒯_*i*_ = the 91 edges within patient *i*, and *F*_*i*_ is the distribution of edge weights within that patient’s network. This within-patient formulation treats each individual’s 91 edge weights as evaluations of their network organization function, computing how typical each edge is relative to that patient’s overall connectivity architecture. We acknowledge that alternative multivariate approaches (e.g., Mahalanobis depth, projection depth) could be applied directly to the 91-dimensional vectors. However, the FM depth’s explicit integration over the domain (edge index) provides interpretable contributions from individual pathway pairs, facilitating biological interpretation. The computational cost of FM depth (*O*(*n*^2^ *p*) for *n* patients and *p* edges) remains tractable for our dataset (*n* = 996, *p* = 91).

### 4.6 Justification of Dual Depth Framework

Our framework employs two complementary reference frames grounded in distinct precision oncology paradigms. Patient Referenced Depth operationalizes N-of-1 medicine by measuring pathway network organization relative to each individual’s molecular baseline. This approach finds strong support from prospective trials demonstrating superiority of individualized molecular profiling: the I-PREDICT trial showed patients with therapies matched to their specific alterations had improved outcomes^19^, while molecular tumor board analyses across thousands of patients confirmed better outcomes when treatments target individual molecular landscapes^68,69^.

Computational methods for single subject analysis provide methodological precedent. The N-of-1-pathways framework established that comparing paired samples within individuals detects personal deregulated mechanisms^70–73^. Frost demonstrated that individual tumor dysregulation scores computed relative to patient specific baselines outperform population comparisons^21,31^. Our Patient Referenced Depth extends these concepts to graph structured pathway networks.

Population Referenced Depth identifies patients whose molecular profiles deviate from cohort norms, a strategy validated by cancer outlier detection methods. Transcriptome outlier analysis reveals that systematic deviations from population distributions pinpoint therapeutic vulnerabilities^74^, while functional genomic outliers are enriched for actionable targets^75^. Multiple approaches applied to lung cancer confirm that population referenced deviations identify prognostically relevant patterns^76,77^. These findings rest on functional data depth theory, where depth measures quantify observation centrality within population distributions^25,35,78–80^.

The limited feature overlap demonstrates complementary information. Patient Referenced Depth identifies coordination patterns within individual architectures, aligning with molecular tumor board paradigms^81–83^. Population Referenced Depth identifies systematic subtype deviations, analogous to basket trial enrollment^84^. The superior classification performance of Patient Referenced Depth validates that individual optimization outperforms population stratification, while Population Referenced Depth remains valuable for initial classification and outlier identification^85,86^.

### 4.7 Random Forest Classification

We trained random forest models using the ranger and randomForest R packages^87–89^ with 5 fold cross validation repeated 3 times for hyperparameter tuning. Final models used 1500 trees with permutation based importance. Class weights were set to inverse class frequency to handle class imbalance. Performance metrics included AUC-ROC, accuracy, sensitivity, and specificity. We determined optimal classification threshold using Youden’s index on training set ROC curves.

### 4.8 Rule Extraction from Random Forest Model

To enhance model interpretability and identify discriminatory patterns between LUAD and LUSC subtypes, we extracted explicit decision rules from the trained random forest classifiers. After training on Population Referenced Depth (across patient functional depth features) and Patient Referenced Depth (within patient functional depth features), we rebuilt the optimized random forests using the randomForest package with hyperparameters identified during cross validation (mtry, number of trees, and minimum node size).

#### 4.8.1 Decision Rule Extraction

We extracted decision rules from the first 20 trees of each random forest model, yielding 2,757 unique rules for Population Referenced Depth and 1,479 unique rules for Patient Referenced Depth (100% rule uniqueness). Feature coverage analysis confirmed that rules from these 20 trees captured all top 50 most important features and 98.9–100% of the entire feature space (90–91 of 91 pathway interaction features), justifying this 20 tree size as sufficient for comprehensive rule-based interpretation.

For each model, we extracted decision rules from the first 20 trees using a depth first traversal algorithm. The extraction procedure identified all root to leaf paths within each tree, recording the sequence of splitting conditions (feature thresholds) and terminal node predictions. Each extracted rule consists of: (1) a conjunction of splitting conditions defining the decision path, (2) the predicted class label at the terminal node, and (3) the rule complexity measured by the number of conditions. This rule based representation provides explicit logical statements describing how depth features collectively discriminate between LUAD and LUSC samples, making biological interpretation easier beyond standard variable importance metrics. We cataloged the extracted rules separately for Population Referenced Depth and Patient Referenced Depth models to compare the discriminatory patterns identified through across patient versus within patient functional variability.

#### 4.8.2 Depth First Traversal Algorithm

The depth first traversal algorithm systematically extracts all decision paths from each random forest tree by recursively exploring the tree structure from root to leaves^90–96^. Starting at the root node, the algorithm examines whether the current node is a terminal (leaf) node or an internal decision node. At each internal node, the algorithm identifies three components: (1) the splitting feature, (2) the threshold value, and (3) the left and right child nodes corresponding to samples satisfying feature ≤ threshold and feature > threshold, respectively. The algorithm maintains a growing vector of conditions as it descends the tree. When encountering an internal node with split condition “*X*_feature_ ≤ *c*”, the algorithm first recursively explores the left subtree, appending “*X*_feature_ ≤ *c”* to the condition vector, then backtracks to explore the right subtree with “*X*_feature_ > *c”* appended instead. This depth first strategy ensures complete exploration of all paths before moving to alternative branches.

When reaching a leaf node, the algorithm records the complete decision rule as the conjunction of all accumulated conditions along the path from root to leaf, paired with the leaf’s class prediction (LUAD or LUSC). The traversal continues until all root to leaf paths have been enumerated. A maximum depth parameter prevents infinite recursion in case of corrupted tree structures, and nodes lacking valid split information are skipped. This approach guarantees extraction of every possible classification rule encoded in the tree structure, capturing both simple shallow rules and complex deep rules involving multiple feature interactions.

### 4.9 Differential Expression Analysis

To identify pathway interactions that exhibit distinct depth patterns between LUAD and LUSC, we performed differential expression analysis on both Population Referenced Depth (population-level) and Patient Referenced Depth (within-patient) representations using the training dataset. For each pathway feature, we compared depth values between the LUSC (Control) and LUAD (Disease) groups using Welch’s two-sample t-test, which does not assume equal variances between groups. We calculated the effect size using Cohen’s D:$=\frac{{\overset{‾}{x}}_{\text{LUAD}}-{\overset{‾}{x}}_{\text{LUSC}}}{s_{\text{pooled}}}$ where ${\overset{‾}{x}}_{\text{LUAD}}$ and ${\overset{‾}{x}}_{\text{LUSC}}$ are the mean depth values for each group, and *s*_pooled_ is the pooled standard deviation: $=\sqrt{\frac{\left(n_{\text{LUAD}}-1\right)s_{\text{LUAD}}^{2}+\left(n_{\text{LUSC}}-1\right)s_{\text{LUSC}}^{2}}{n_{\text{LUAD}}+n_{\text{LUSC}}-2}}$. Cohen’s D provides a standardized measure of effect size independent of sample size, with |*D*| > 0.2, |*D*| > 0.5, and |*D*| > 0.8 typically representing small, medium, and large effects, respectively. To control for multiple testing, we adjusted p-values using the Benjamini-Hochberg false discovery rate (FDR) procedure. Features with FDR-adjusted p-values below 0.05 were considered statistically significant. We also computed log2 fold change as: $={\text{log}}_{2}\left(\frac{{\overset{‾}{x}}_{\text{LUAD}}+1e^{-100}}{{\overset{‾}{x}}_{\text{LUSC}}+1e^{-100}}\right)$ where a small constant (1*e*^−100^) was added to prevent division by zero. We visualized the results using volcano plots, with Cohen’s D on the x-axis and −log_10_(FDR) on the y-axis. Features were categorized as “Strong Effect” (FDR < 0.05 and |Cohen’s D| > 0.3), “Significant” (FDR < 0.05 and |Cohen’s D| ≤ 0.3), or “Not Significant” (FDR ≥ 0.05). This classification highlights pathway interactions with both statistical significance and meaningful biological effect sizes. The top 15 features by absolute Cohen’s D were displayed separately, stratified by whether they showed higher depth values in LUAD or LUSC, revealing the directional patterns of pathway dysregulation in each cancer subtype.

### 4.10 Feature Co-occurrence Analysis

To identify functional depth features that collectively contribute to subtype discrimination, we performed pairwise feature co-occurrence analysis on the extracted decision rules from the first 20 trees. For each decision rule, we identified all depth features appearing in the conjunctive conditions using regular expression pattern matching. We then computed the frequency of pairwise co-occurrences across all rules within each model.

Specifically, for rules containing two or more features, we enumerated all unique feature pairs and tallied their joint appearances across the rule set. We built feature co-occurrence networks by connecting features appearing together in rules, with edge weights representing co-occurrence frequency. We characterized network topology using degree centrality, betweenness centrality, and clustering coefficient. A high co-occurrence frequency indicates that two depth features are consistently used together in the same decision paths, suggesting potential functional or biological interactions relevant to LUAD versus LUSC classification. We conducted this analysis independently for Population Referenced Depth and Patient Referenced Depth models to compare collaborative feature patterns between the across patient and within patient depth representations. The resulting co-occurrence matrices reveal which depth features operate synergistically in the classification mechanism, providing insight into coordinated functional variability patterns that distinguish the two lung adenocarcinoma subtypes beyond univariate feature importance rankings.

### 4.11 Feature importance and cross method integration

To identify the most informative pathway interactions for LUAD versus LUSC classification, we extracted feature importance scores from the trained random forest models using permutation importance. This approach measures the decrease in model performance when each feature’s values are randomly permuted, quantifying each pathway’s contribution to predictive accuracy. For both Population Referenced Depth and Patient Referenced Depth representations, we ranked all pathway features by their permutation importance scores and identified the top 30 features. To assess consistency between the two Population Referenced Depthpproaches, we compared the top 15 features from each method and quantified their overlap. This analysis reveals whether population-level depth patterns (Population Referenced Depth) and within-patient depth patterns (Patient Referenced Depth) identify similar or distinct pathway signatures. We integrated random forest feature importance with differential expression analysis to identify pathway interactions that are both statistically significant and predictively powerful. For each feature, we combined two complementary rankings: (1) rank by absolute Cohen’s D effect size from differential expression testing, and (2) rank by random forest permutation importance. The combined score was calculated as Combined ${\text{Score}}_{i}=\frac{1}{2}\left(\frac{1}{{\text{Rank}}_{\text{Diff},i}}+\frac{1}{{\text{Rank}}_{\text{Imp},i}}\right)$ where higher scores indicate features that rank highly by both criteria. This multi-method integration prioritizes pathway interactions that show strong biological differences between cancer subtypes (high Cohen’s D) while also contributing substantially to classification performance (high RF importance). We visualized the relationship between differential expression and predictive importance through scatter plots, with point size scaled by the combined score. Features with FDR-adjusted p-values below 0.05 were colored to highlight statistically significant pathway interactions. This integrative approach identifies robust biomarker candidates that satisfy both statistical significance and predictive utility criteria, reducing the likelihood of selecting spurious features that excel in only one dimension.

### 4.12 Integrated Multi-Method Analysis

We integrated results across methods by identifying features meeting multiple criteria: (1) Top 30 by random forest importance, (2) Top 75th percentile by absolute PC1 loading, (3) Significantly differential (FDR < 0.05). Features meeting all three criteria were considered robust biomarker candidates.

### 4.13 Statistical Analysis

All analyses were performed in R version 4.5.1. Depth computation used the fda.usc package^67^. Random forest modeling used randomForest and ranger packages^88,89^. A significance level of *α* = 0.05 was used throughout with multiple testing correction where appropriate.

## Figures and Tables

**Table 1.** Performance metrics for Population Referenced Depth and Patient Referenced Depth random forest models. Patient Referenced Depth (within patient network organization) outperforms Population Referenced Depth (population level typicality) across multiple metrics.

Model	Type	AUC	Accuracy	Sensitivity	Specificity	F1-score
Population Referenced Depth	Training	1.0000	1.0000	1.0000	1.0000	1.0000
	Test	0.7505	0.6910	0.7520	0.6240	0.7200
	OOB	0.7527	0.7822	—	—	—
PatientReferenced Depth	Training	1.0000	1.0000	1.0000	1.0000	1.0000
	Test	0.7681	0.7080	0.6750	0.7450	0.7090
	OOB	0.7556	0.7947	—	—	—

## Data Availability

All data are publicly available through The Cancer Genome Atlas (TCGA) database and DeepSpot^62^.

## Code Availability

Code implementing the functional depth analysis pipeline may be made available to researchers on reasonable request.
